# OsPRD2 is essential for double-strand break formation, but not spindle assembly during rice meiosis

**DOI:** 10.3389/fpls.2022.1122202

**Published:** 2023-01-13

**Authors:** Chong Wang, Shuying Qu, Jie Zhang, Ming Fu, Xiaofei Chen, Wanqi Liang

**Affiliations:** ^1^ Joint International Research Laboratory of Metabolic & Developmental Sciences, State Key Laboratory of Hybrid Rice, School of Life Sciences and Biotechnology, Shanghai Jiao Tong University, Shanghai, China; ^2^ Development Center of Plant Germplasm Resources, Shanghai Key Laboratory of Plant Molecular Sciences, College of Life Sciences, Shanghai Normal University, Shanghai, China

**Keywords:** OsPRD2, meiosis, DSB, spindle, centromere

## Abstract

Meiotic recombination starts with the programmed formation of double-strand breaks (DSB) in DNA, which are catalyzed by SPO11, a type II topoisomerase that is evolutionarily conserved, and several other accessary proteins. Homologs of MEIOSIS INHIBITOR 4 (MEI4/REC24/PRD2) are proteins that are also essential for the generation of meiotic DSBs in budding yeast, mice and Arabidopsis thaliana. In Arabidopsis, the protein ARABIDOPSIS THALIANA PUTATIVE RECOMBINATION INITIATION DEFECTS 2/MULTIPOLAR SPINDLE 1 (AtPRD2/MPS1) has been shown to have additional roles in spindle assembly, indicating a functional diversification. Here we characterize the role of the rice MEI4/PRD2 homolog in meiosis. The *osprd2* mutant was completely male and female sterile. In male meiocytes of *osprd2*, no γH2AX foci were detected and twenty-four univalents were produced at diakinesis, suggesting that OsPRD2 is essential for DSB generation. OsPRD2 showed a dynamic localization during meiosis. For instance, OsPRD2 foci first appeared as discrete signals across chromosome at leptotene, and then became confined to the centromeres during zygotene, suggesting that they might be involved in assembly of the spindle. However we did not observe any obvious aberrant morphologies in neither the organization of the bipolar spindle nor in the orientation of the kinetochore in the mutant. These findings suggest that in rice PRD2 might not be required for spindle assembly and organization, as it does in Arabidopsis. Taken together our results indicate that plant MEI4/PRD2 homologs do play a conserved role in the formation of meiotic DSBs in DNA, but that their involvement in bipolar spindle assembly is rather species-specific.

## 1 Introduction

Meiosis is a specialized cell division in eukaryotes, during which one round of DNA replication followed by two rounds of cell division, producing haploids with halved DNA content. Homologous chromosomes established physical links *via* recombination to ensure accurate separation of parental information. Meiotic recombination depends on the double stand breaks (DSBs) formation, which are produced by the topoisomerase VI (TOPVI) protein complex and several accessary proteins. The topoisomerase VI complex is evolutionarily conserved, composed of the catalytic A subunit Spo11 and the B subunit MTOPVIB (Fu et al., 2016; Vrielynck et al., 2016; Xue et al., 2016). In contrast, accessory proteins are variable in different species.

In *Saccharomyces cerevisiae* (*S. cerevisiae*), nine accessary proteins were found to act together with Spo11 to generate DSBs, including Ski8, Rec114, Mei4, Mer2, Rec102, Rec104, Mre11, Rad50 and Xrs2 (Lam and Keeney, 2014). In Arabidopsis, PUTATIVE RECOMBINATION INITIATION DEFECT 1 (AtPRD1), AtPRD2/Multipolar spindle1 (MPS1) and AtPRD3 are indispensable for DSB formation (De Muyt et al., 2009). They have been suggested to be the ortholog of Mei1 in mice, Mei4 in yeast and Mer2 in yeast respectively (Cole et al., 2010; Tesse et al., 2017), although they display enormous sequence divergence with their orthologs in yeast or mice. Another accessary protein, Arabidopsis thaliana DSB formation (AtDFO), is a plant-specific protein and no homolog has been identified outside the plant kingdom (Zhang et al., 2012). In rice, in addition to functionally conserved OsPRD1, four other DSB proteins have been identified, namely CENTRAL REGION COMPONENT1 (CRC1), SOLO DANCERS (OsSDS), P31^comet^/Bivalent Formation 1 (OsBVF1) and RNA-dependent RNA polymerase 6 (OsRDR6) (Miao et al., 2013; Wu et al., 2015; Ji et al., 2016; Zhou et al., 2017; Liu et al., 2020). Intriguingly, although the homologs of OsSDS and OsCRC1 in other organisms play important roles in meiosis (Bhalla and Dernburg, 2005; Joyce and Mckim, 2009; Zanders and Alani, 2009; Roig et al., 2010), they are not required for DSB formation. Furthermore, the homolog of P31^comet^/OsBVF1 in human was shown to be involved in the spindle assembly checkpoint in mitosis (Yang et al., 2007). RDR6 is a key component of the small RNA biogenesis pathway. Mutations in rice RDR6 abolish meiotic DSB formation, however whether its homologs in other species have the same role is yet unknown (Liu et al., 2020). These studies suggest that the Spo11 accessory proteins are diversified at the sequence or functional level across the eukaryotes.

Mei4 is one of the nine accessary proteins in *S. cerevisiae* which was first found by screening for mutants defective in meiotic recombination (Menees and Roeder, 1989). In *S. cerevisiae*, Rec114, Mei4 and Mer2 form a stable functional complex (the RMM complex) to promote DSB formation by connecting the DSB machinery to the chromosome axis (Maleki et al., 2007). A recent study indicated that DNA-driven RMM condensates may regulate DSB repair by nucleating formation of the recombination nodules at repair sites, which may control DSB number, location as well as timing, and coordinate DSB formation with downstream repair during meiosis (Claeys et al., 2021). Mei4 homologs in mouse and Arabidopsis have been demonstrated to have conserved roles in DSB formation (Kumar et al., 2010). In Arabidopsis, AtPDR2/MPS1 is also involved in meiotic spindle organization during meiosis (Jiang et al., 2009; Walker et al., 2018). In rice, it has been shown that OsPRD2 formed a complex with OsPRD1 and OsMTOPVIB (Shi et al., 2021). It is likely that OsPRD2 may participate in both DSB formation and spindle assembly during rice meiosis, while its function in rice meiosis has not been investigated.

In this study, we isolated the rice *osprd2* mutant and demonstrated that OsPRD2 is essential for DSB formation. We found that meiotic DSBs were absent in *osprd2* male meiocytes and 24 univalents were formed at diakinesis. In addition, albeit OsPRD2 was localized in the centromere region at zygotene, no significant defects in the spindle assembling was observed in *osprd2*, which is different from *mps1*/*prd2* mutants in Arabidopsis. Our results indicate that in rice OsPRD2 has a conserved role in meiotic DSB formation, but it does not participate in bipolar spindle assembly during meiosis I, suggesting a functional divergence among different plant species.

## 2 Materials and methods

### 2.1 Plant materials and growth conditions

The *osprd2* mutant and other mutant used in this study were 9522 background (*O. sativa* ssp. *japonica*). For map-based clone, the other parent was 9311 (*O. sativa* ssp. *indica*). All the mutant plant and transgenic plants were grown in paddy field and rice culture room of Shanghai Jiao Tong University. The trans-genetic plants were growth in rice growth chamber in Shanghai Jiao Tong University.

### 2.2 Mutant phenotypic analyses

The whole plant materials were photographed by a digital camera (Nikon; catalog no. E995) and floral organs were photographed with a dissecting microscope (Motic; catalog no. K400). DAPI staining, Immunolocalization assays and FISH assays were analyzed as reported (Wang et al., 2017; Zhang et al., 2019) and photographed using the Eclipse Ni-E microscope (Nikon).

### 2.3 Spindle structure observation

Spindle structure were observed by the methods modified from previously described (Li et al., 2010). Fresh rice meiosis infloresence were fixed in 4% paraformaldehyde solution and followed by 10%DMSO and 1% TritonX-100 post-processing. Anthers at suitable meiosis stages were isolated and digested for 30 minutes in enzyme solutions.

Then anther were broken to release meiocytes on slides. After dry in air, tubulin antibody were diluted by 1:100 and incubated overnight. Wished by 1×PBS for three times and incubated with FITC labored second antibody in 37 °C for 1 hour. Then slides were wished again in 1×PBS and added DAPI for observation.

### 2.4 Antibody production

For obtaining OsPRD2 polyclonal antibody, a 900-bp DNA fragment starting from ATG which encoding N terminal 300 amino acid peptide of OsPRD2 was amplified from rice anther cDNA and cloned into pGEX-4T-1 (GE). The recombinant protein was expressed in Escherichia coli DE3 (BL21; Novagen) and purified to produce rat and rabbit polyclonal antibodies (prepared by Abclonal) specific to OsPRD2 (**Figure S4**). Primers used for *OsPRD2* fragment amplification and construction were listed in **Supplementary Table 1**. Antibody against OsSGO1, PAIR2, PAIR3, ZEP1, γH2AX, DMC1, HEI10 and CENH3 were generated as reported (Wang et al., 2011; Fu et al., 2016; He et al., 2016; Wang et al., 2017; Zhang et al., 2019). Tubulin antibody were purchased from Beyotime (AT819) and H3S10 antibody were obtain from sigma-aldrich (05-806).

### 2.5 CRISPR knockout of *OsSGO1*

The CRISPR vector for OsSGO1 knockout were constructed as previously described (Xie et al., 2015). Constructed OsSGO1 CRISPR plasmids was transformed into *A.tumefaciens* (EHA105) and infected rice calli as reported (Hiei and Komari, 2008). The primers for constructing CRISPR vectors and *ossgo1* mutant identification were listed in **Supplementary Table 1**.

### 2.6 Phylogenetic analysis

The protein sequences were aligned using ClustalX (Larkin et al., 2007), and then adjusted manually using GeneDoc software (http://www.psc.edu/biomed/genedoc/). Neighbor-joining trees were constructed using the MEGA (version 4.0) software (Tamura et al., 2007) with the following parameters: Poisson correction, pairwise deletion, and bootstrap (1,000 replicates; random seed).

## 3 Results

### 3.1 Isolation of *osprd2*

To understand the mechanisms underlying rice fertility control, we isolated a completely sterile mutant from our rice mutant library (Chen et al., 2006) and designated *osprd2*, as the mutation occurred in the rice homolog of Arabidopsis *PRD2*/MPS1 (See below). Compared with wild-type plants, *osprd2* showed no obvious differences during vegetative growth, and displayed normal panicle and spikelet morphology (**Figure S1 A, B, D, E**). *osprd2* could not produce fertile pollen (**Figure S1 G, H**) and set no seeds when pollinated with wild-type pollen grains, indicating that *osprd2* was defective in both male and female fertility. We crossed *osprd2* heterozygotes with wild type. All F1 plants had normal fertility and in the F3 populations that segregated sterile plants, the segregation rate was 3:1 (χ2 = 0.169 < χ0.052 = 3.84). All the results showed that *osprd2* sterility was caused by a single recessive genic mutation.

**Figure 1:**
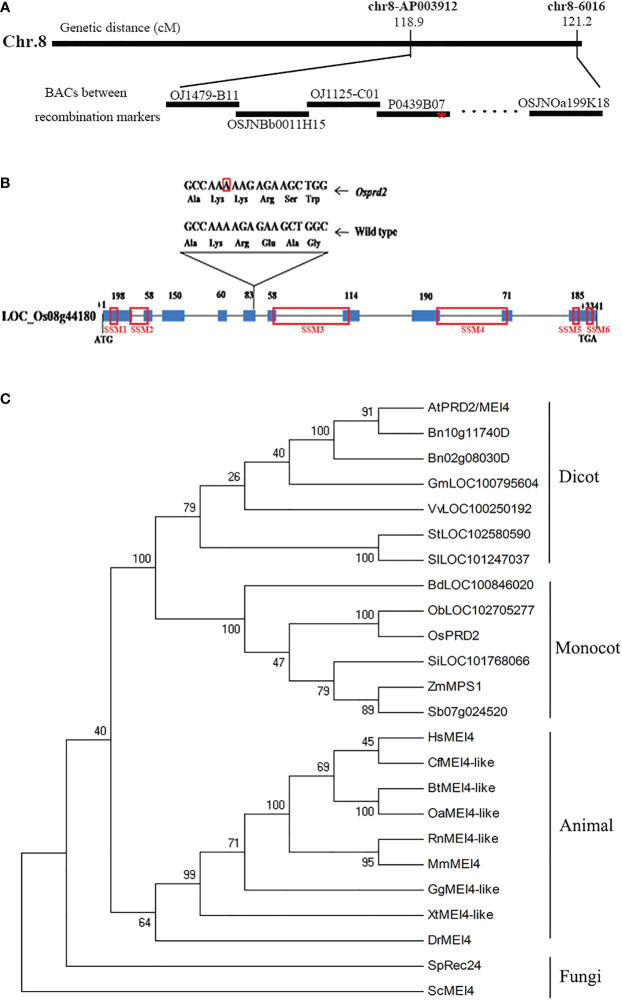
Map-based cloning of *OsPRD2* and phylogenetic analysis of proteins homologous to OsPRD2. **(A)** Map-based cloning of *OsPRD2* gene, red star indicated the mutation site; **(B)** gene structure and the mutated sites of *OsPRD2*, the nucleic acid with red box indicated A insertion in *osprd2* mutant, red box on the gene indicated the conserved SSMs motif of PRD2/MEI4 family members; **(C)** neighbor-joining tree of proteins homologous to OsPRD2. The tree was constructed from the alignment of full-length proteins in rice and other species. The unannotated sequences accession numbers are given in the brackets after species names.

To identify the causative mutation gene, we employed the map-based cloning approach using a mapping population constructed by crossing *osprd2* heterozygotes (*japonica* cultivar 9522) with wild-type plants (*indica* cultivar 9311). The mutation was mapped to a region on chromosome 8 between the molecular markers OJ1479-B11 and OSJNOa199K18, which is covered by 11 Bacterial Artificial Chromosome clones (BACs) and the genetic distance was 2.3cM (**Figure 1A**). We then performed high-through put sequencing analysis and found one base pair insertion in the fifth exon of LOC_Os08g44180, which encodes a protein sharing high sequence similarity with AtPRD2. The 1bp insertion led to frameshift, generating a premature translation stop codon after 237 amino acid and a truncated mutant protein lacking approximately half of the protein sequence (**Figure 1B**). The fertility of *osprd2* was recovered by a wild-type genomic DNA fragment containing the coding region, 3.8 Kb upstream sequence and 2.2 Kb downstream sequence of *OsPRD2* (**Figure S1 C, F, I**), confirming that the mutation was responsible for the sterility of *osprd2*.

The full-length OsPRD2 protein sequence was used as a query to perform Position-Specific Iterative Basic Local Alignment Search Tool (PSI-BLAST) homology searches. PRD2 is widely conserved and its homologs are found in fungi, animals and plants. We built a neighbor-joining tree using 24 sequences from 23 species. OsPRD2 was divided into the monocot plants branch (**Figure 1C**). Sequence alignment revealed that OsPRD2 contained 6 conserved signature sequence motifs (SSMs) that were predicted to adopted α-helical or coiled-coiled structures and might be involved in protein-protein interactions (Kumar et al., 2010).

### 3.2 Disruption of *OsPRD2* causes meiotic defects

**Figure 2:**
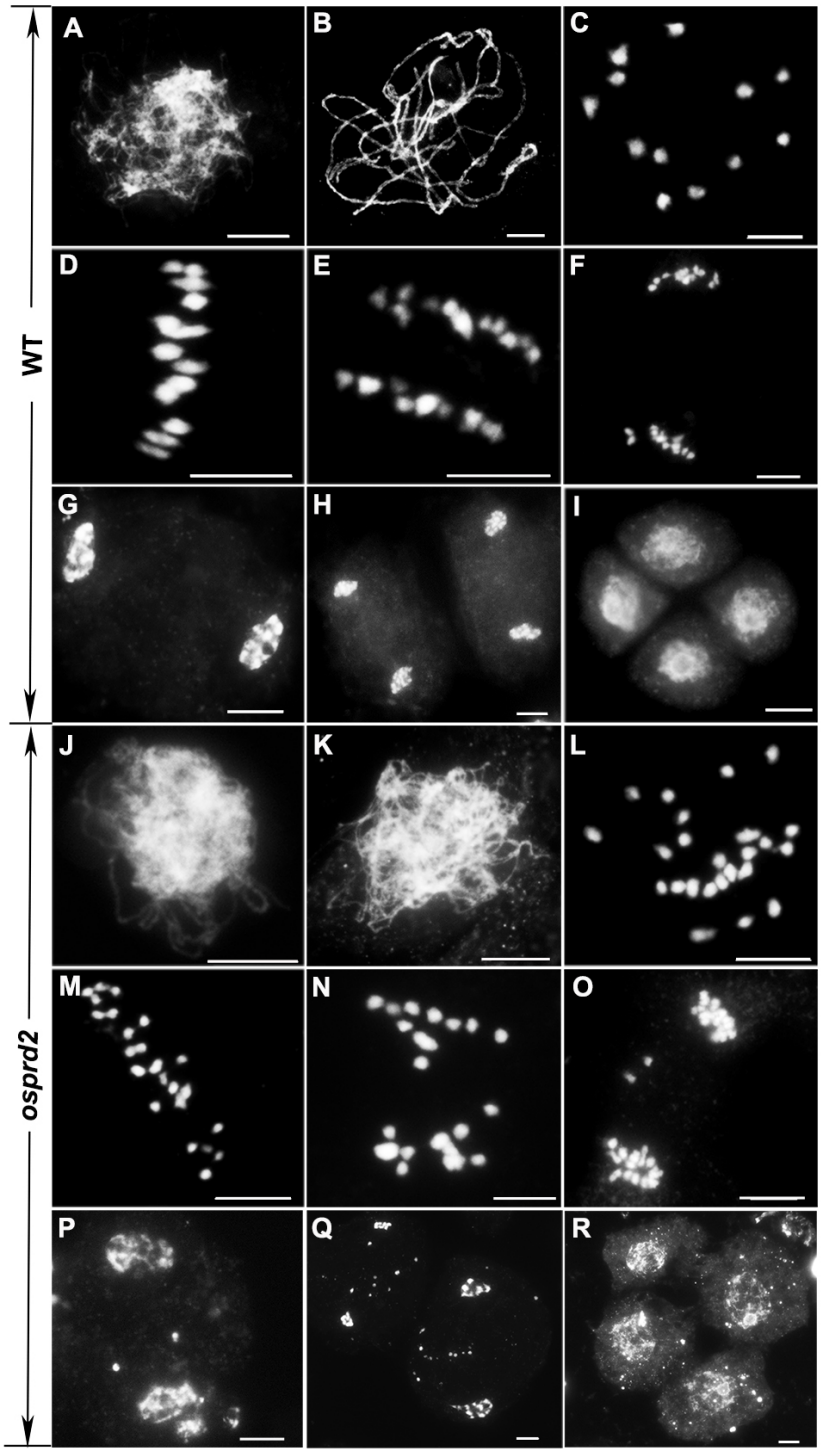
Meiotic chromosome behavior in WT and *osprd2*. **(A, J)** leptotene, **(B, K)** pachytene, **(C, L)** diakinesis, **(D, M)** metaphase I, **(E, K)** anaphase I, **(F, O)** telophase I, **(G, P)** prophase II, **(H, Q)** telophase II, **(I, R)** tetrad. **(A-I)** are wild type; **(J-R)** are *osprd2* mutant. Bar=5μm.

To investigate the potential role of *osprd2* in meiosis, we monitored the meiosis progression in *osprd2* by 4’,6-diamidino-2-phenylindole (DAPI) staining. In the wild-type, chromosomes began to condense and become visible as thin threads at leptotene (**Figure 2A**). At zygotene, homologous chromosomes started to pair with each other and synapsis initiation sites formed. At pachytene, homologous chromosomes fully synapsed with formation of the synaptonemal complex (SC) (**Figure 2B**). Twelve bivalents appeared at diakinesis, which then aligned along the equatorial plate at metaphase I (**Figures 2C, D**). The homologous chromosomes then separated and were pulled toward the opposite poles by the spindle from anaphase I to telophase I (**Figures 2E, F**). During the second meiotic division, sister chromatids of each chromosome segregated and tetrads formed at the end of meiosis II (**Figures 2G-I**). In *osprd2*, male meiocytes exhibited normal configuration at leptotene (**Figure 2J**). However, homologous chromosomes did not paired and synapsed (**Figure 2K**), leading to generation of 24 unpaired univalents at diakinesis (**Figure 2L**). An later stages, the univalents randomly separated to two poles (**Figures 2M-P**). During meiosis II, sister chromatids were randomly separated (**Figure 2Q**). As a result, abnormal tetrads with multiple micronuclei were produced at the end of meiosis (**Figure 2R**). These results indicated that the male sterility of *osprd2* was caused by defects in meiosis.

### 3.3 *OsPRD2* is required for meiotic DSB formation

**Figure 3:**
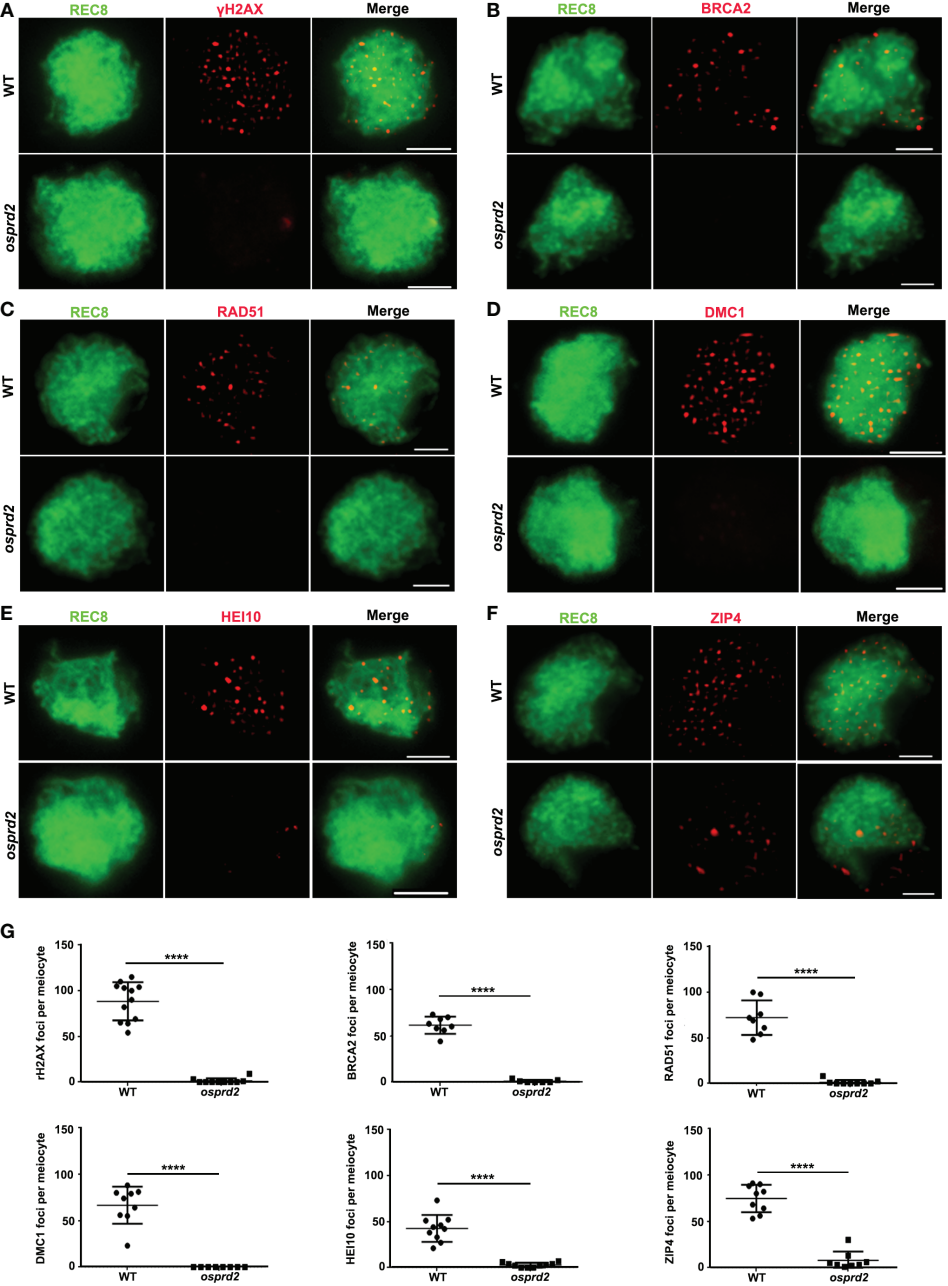
Immunostaining detection of homologous recombination related proteins in the WT and *osprd2* mutant. **(A)** is γH2AX signals (red) at zygotene; **(B)** is BRCA2 signal (red) at zygotene; **(C)** is RAD51 signals (red) at zygotene; **(D)** is DMC1 signals (red) at zygotene; **(E)** is HEI10 signals (red) at zygotene; **(F)** is ZIP4 signals (red) at zygotene; OsREC8 signals (green) were used to indicate the meiotic chromosome axes;**(G) **Statistical analysis of the number of γH2AX, BRCA2, RAD51, DMC1, HEI10 and ZIP4 foci per meiocyte in the wild type and *osprd2*. error bars indicate SD, ****p < 0.0001, t tests. Bar=2μm.

The univalent formation indicated that *osprd2* may be defective in homologous chromosome pairing, synapsis or recombination. To further understand the role of *OsPRD2* in meiosis, we investigated the impact of the *OsPRD2* mutation on these events. It has been reported that OsPRD2 orthologs play important roles in meiotic DSB generation in yeasts, mice, mouse and Arabidopsis (Bonfils et al., 2011; Kumar et al., 2010; Kumar et al., 2015; Martin-Castellanos et al., 2005). We thus first analyzed DSB formation using rH2AX as a DSB marker. Compared with wild type, there were no rH2AX signals during early prophase I in *osprd2* (**Figure 3A**), indicating no DSB formation in *osprd2*. As a result, DSB repair and homologous recombination related proteins, such as BRCA2, RAD51, DMC1, HEI10 and ZIP4 could not localize to the meiotic chromosome at zygotene (**Figures 3B-G**).

**Figure 4:**
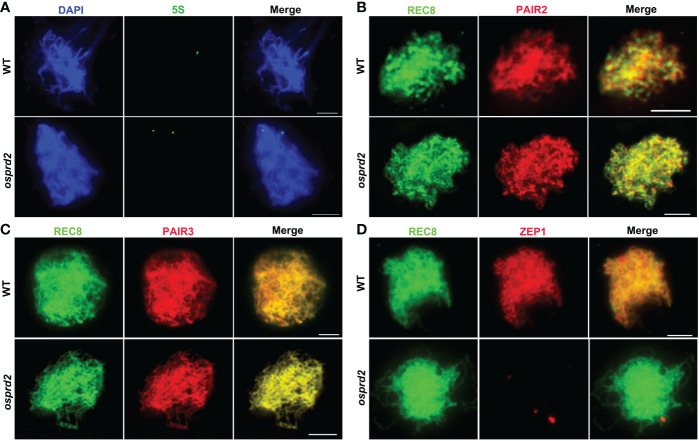
Homologous chromosome pairing and synapsis were disrupted in *osprd2*. **(A)** is 5S probe (green) signal in WT and *osprd2* mutant in zygotene; **(B–D)** are distribution of synapsis complex element PAIR2 **(B)**, PAIR3 **(C)** and ZEP1 **(D)** (red) in WT and *osprd2* mutant in zygotene, REC8 signals (green) were used to indicate the meiotic chromosome axes. Bar=2μm.

We next performed fluorescent *in situ* hybridization (FISH) analysis using a 5S repetitive DNA (rDNA) probe located on the short arm of chromosome 11, Cy3 probe-labeled telomere-specific sequences and a centromere-specific tandem repeat of O. sativa (CentO) probe separately to analyze chromosome pairing progression in *osprd2*. In wild type, telomere clustered together to the nuclear envelope as a bouquet at early prophase I, which brought chromosome into close proximity. In *osprd2*, telomere bouquet was regularly formed which was shown by the telomere probe (**Figure S2**), indicating that the initial stage of chromosome pairing was not affected. However, FISH analysis with the 5S rDNA probe revealed that homologous chromosomes were unable to pair together at later stage (**Figure 4A**). Furthermore, abnormally elongated centromere signals were observed in *osprd2* at zygotene, pachytene and diakinesis (**Figure S3**), suggesting a possible delay in chromosome condensation at the centromere region.

To monitor SC formation in *osprd2* and wild type, we detected immunolocalization of SC elements: axle elements PAIR2, PAIR3 and central element ZEP1. During zygotene, although PAIR2 and PAIR3 normally colocalized with REC8 alongside the chromosomes in *osprd2* (**Figures 4B, C**). On the contrary, no ZEP1 signals were observed in *osprd2* (**Figure 4D**), indicating that SC formation could not completed in *osprd2*. These results indicate that OsPRD2 plays a conserved role in DSB formation during rice meiosis. The defect in DSB formation in *osprd2* disrupts the progression of homologous chromosome pairing, synapsis and recombination.

### 3.4 *osprd2* male meiocytes exhibit normal spindle bipolarity

**Figure 5:**
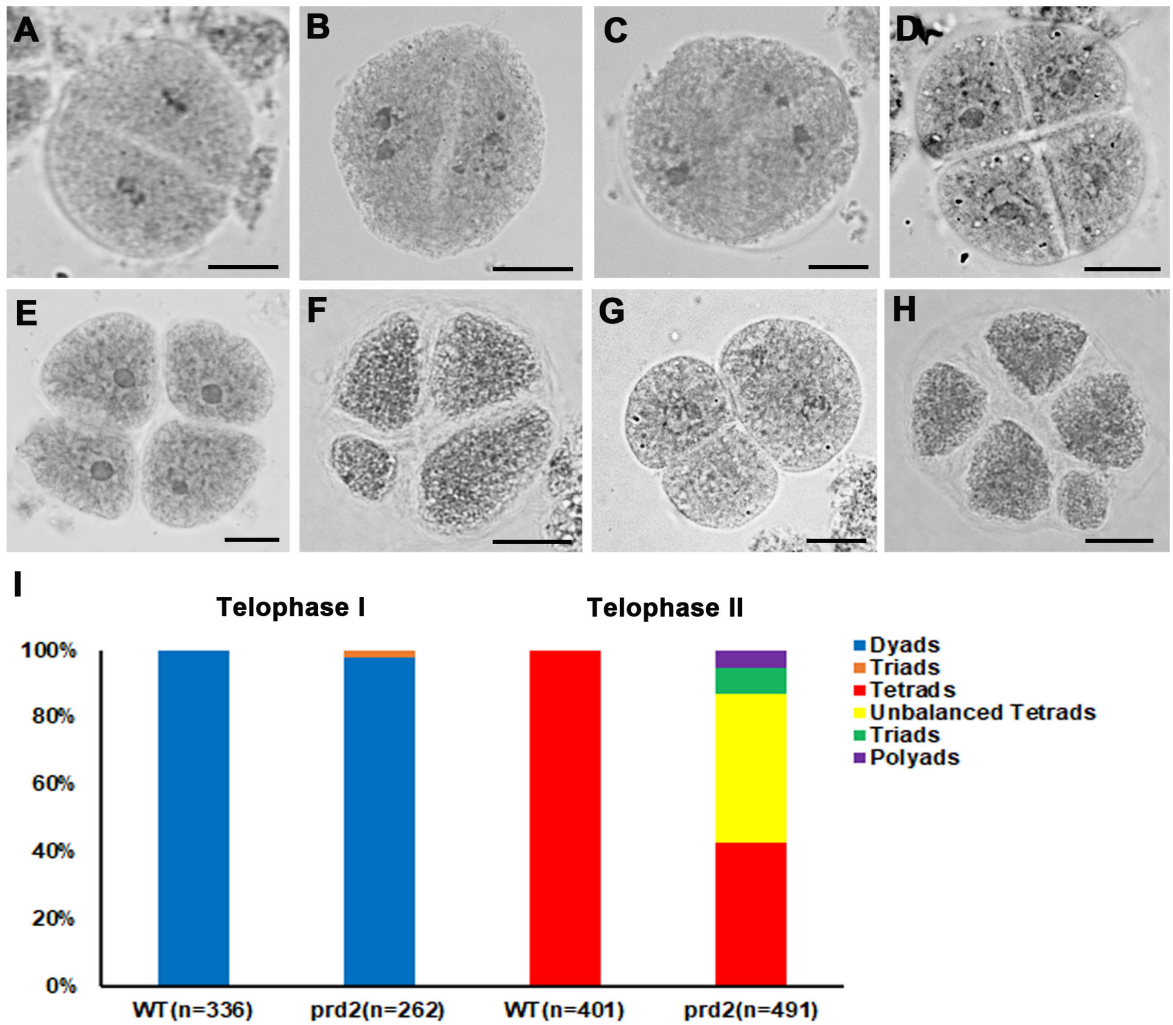
Meiotic products analysis of wild type and *osprd2* mutant. **(A–C)** are telophase I stages; **(D–H)** are telophase II stages; **(A, D)** are wild type; **(B, C, E–H)** are *osprd2* mutant. Bar=5μm.

It has been reported that Arabidopsis OsPRD2 is involved in determining the spindle bipolarity in meiosis. Mutations in *MPS1* cause polyads instead of tetrads (Jiang et al., 2009). In addition, recent studies indicated that in rice OsPRD2 interacts with OsPRD1 and OsMTOPVIB, which are required for both DSB formation and spindle bipolarity (Xue et al., 2019; Shi et al., 2021). To explore the potential role of OsPRD2 in bipolar spindle assembly, we observed the chromosome separation and spindle organization in wild type and *osprd2* using aceto-carmine staining. In wild type, homologous chromosomes were separated and formed dyads in telophase I (**Figure 5A**) and tetrad formation at the end of meiosis II (**Figure 5D**). In contrast, the *osprd2* mutant produced 97.71% dyads and 2.29% triads at telophase I (**Figures 5B, C** and **I**). At telophase II, 43.99% meiocytes shows unbalanced tetrad configuration (**Figures 5E, F** and **I**). In addition, seldom triads (7.94%) and polyads (5.30%) were also found in the *osprd2* mutant (**Figures 5G, H** and **I**).

**Figure 6:**
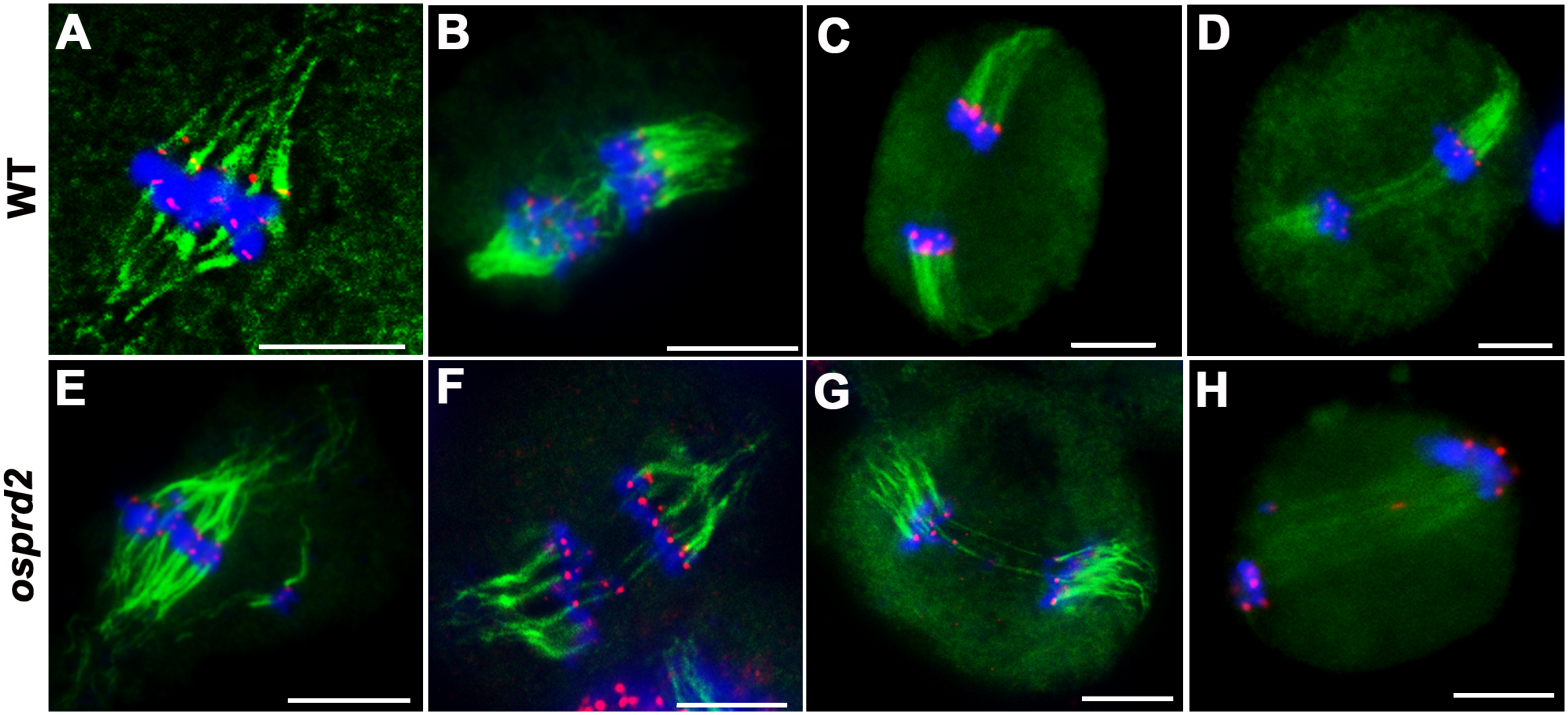
Tubulin structure in wild type and *osprd2* mutant. **(A–D)** are tubulin structure in wild type; **(E–H)** are tubulin structure in *osprd2* mutant. **(A, E)** were metaphase I; **(B, D, F–H)** are anaphase I; green are tubulin signals, red are CENH3 signals and blue indicate chromosome. Bar=5μm.

We next performed dual immunolocalization analysis of CENH3 and microtubule. In wild type, bivalents aligned in the middle of the cell and microtubule fibers were attached to the chromosome centromere regions, by which a bipolar spindle formed at metaphase I (**Figure 6A**). During anaphase I, homologous chromosomes were pulled to two opposite sides (**Figures 6B-D**). In *osprd2*, chromosomes could also link to microtubules and most chromosomes aligned at the middle region of cells during metaphase I (**Figure 6E**). Some univalent distributed randomly with attachment to microtubule filaments. We also observed two centromere signals in some univalents, which indicated premature separation of sister chromatids (**Figure 6F**). At later stages, univalents were separated to the two opposite polar, while a few univalents were lagged at the middle region of the cells and were linked by microtubules with opposite orientations (**Figures 6G, H**). In addition, we did not observe multipolar spindles in the rice *osprd2* mutant at metaphase I, which have been reported in Arabidopsis.

### 3.5 OsPRD2 exhibits dynamic localization during prophase I of meiosis

**Figure 7:**
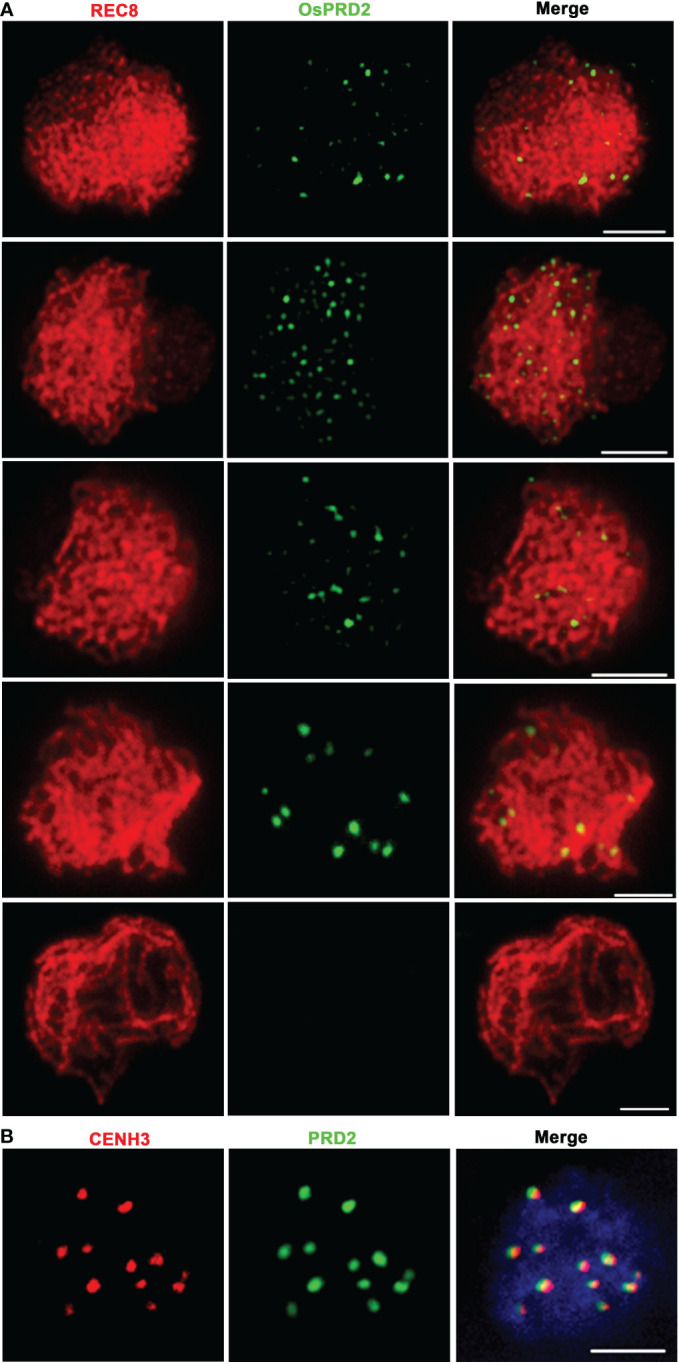
PRD2 co-localized with CENH3 in later meiotic prophase. **(A)** is PRD2 localization during prophase I in wild type; **(B)** is PRD2 and CENH3 co-localization in wild-type zygotene. Bar=2μm.

To further understand its function, dual immunolocalization with polyclonal antibodies against REC8 and a N terminal 300 amino acid peptide in OsPRD2 were conducted to investigate the spatial and temporal distribution of PRD during meiosis. Punctate OsPRD2 foci began to appear and localize on meiotic chromosomes at leptotene, which was consistent with its role in DSB formation. Highest punctate signals were observed at early zygotene (**Figure 7A**). At late zygotene, OsPRD2 foci accumulated into large bright dots (**Figure 7A**) and nearly twelve signal dots were observed. To confirm whether OsPRD2 localize on the centromere at this stage, dual immunolocalization analysis was perform using antibodies against OsPRD2 and the rice centromere maker protein CENH3. The results showed OsPRD2 and CENH3 colocalized from late zygotene to early pachytene (**Figure 7B**), confirming that OsPRD2 localized to centromere at these stages. The OsPRD2 foci disappeared when homologous chromosomes were fully paired (**Figure 7A**). In *osprd2*, no OsPRD2 signal were observed, indicating the antibody was specific for OsPRD2 detection (**Figure S4**).

**Figure 8:**
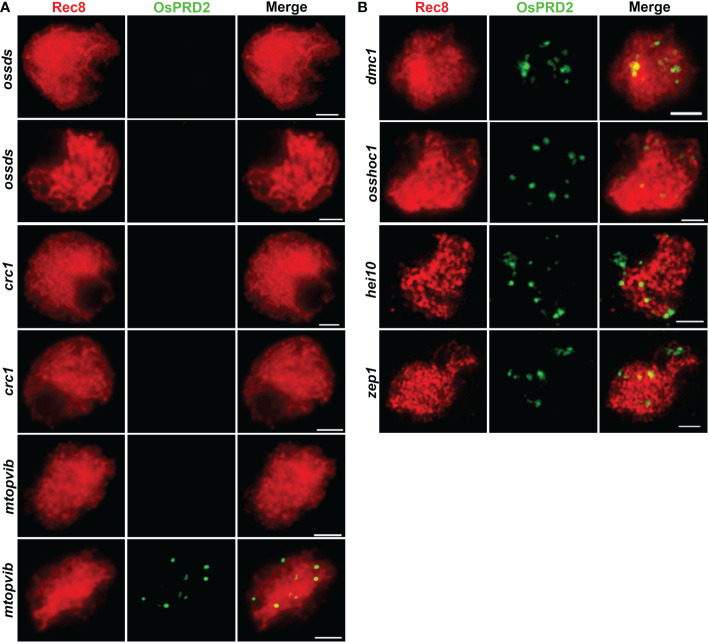
PRD2 localization is dependent on DSB formation but synapsis and recombination. **(A)** is PRD2 localization in mutants defective in DSB formation; **(B)** is PRD2 localization in mutants of key genes involved in synapsis and recombination. Bar=2 μm. **(B)** is informative. PRD2 is required for DSB formation, so it is of course likely that its localization is normal in mutant defective for meiosis at later stages, for example, hei10, which catalyzes CO formation.

To investigate which meiosis processes OsPRD2 participate in, we tested OsPRD2 localization in several rice meiotic mutants, including *ossds*, *crc1*, *mtopVIB*, *dmc1*, *osshoc1*, *hei10* and *zep1*. OsCRC1 and OsSDS is indispensable for DSB formation in rice (Miao et al., 2013; Wu et al., 2015). OsPRD2 signals could not be detected in *oscrc1* and *ossds* (**Figure 8A**), suggesting that their localization to the meiotic chromosomes depends on DSB generation. Interestingly, the punctate foci but not the centromere localized foci of OsPRD2disappeared in *mtopVIB* (**Figure 8A**). DMC1 is required for single-strand invasion during meiotic recombination following DSB processing (Wang et al., 2016). OsSHOC1 and HEI10 are ZMM components in rice. They formed complexes with other rice ZMM proteins and function in promoting class I CO formation (Wang et al., 2012a; Zhang et al., 2019). ZEP1 is the central element of SC in rice which is indispensable for both homologous chromosome synapsis and recombination (Wang et al., 2010). OsPRD2 localization did not rely on the function of these proteins and displayed similar distribution in their mutants (**Figure 8B**).

### 3.6 Mutation in OsPRD2 does not affects kinetochore function

**Figure 9:**
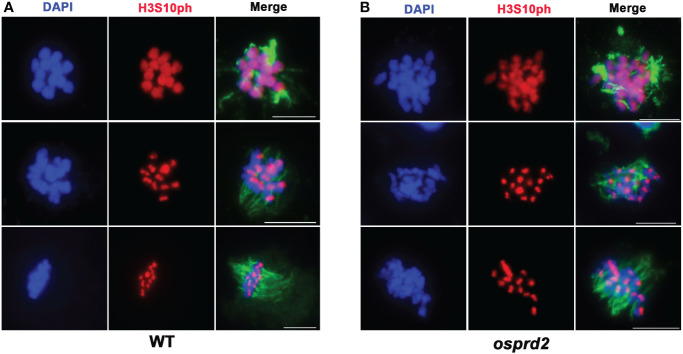
H3S10 signal in wild type and *osprd2* mutant. **(A)** is H3S10 localization in wild type; **(B)** is H3S10 localization in *osprd2* mutant. blue are chromosomes, red are H3S10ph signals and green are tubulin signals. Bar=2μm.

The centromere localization of OsPRD2 suggests that it may play a role in the centromere organization or function. One possibility is that OsPRD2 is required for ensuring proper kinetochore assembly and micro-tubulin fiber attachment. CENH3 was normally loaded at the centromere regions (**Figure 7B**, **Figure S5**), suggesting that the kinetochore assembly was not affected in *osprd2*. Aurora kinases is one of the core spindle checkpoint kinases that are crucial for transducing spindle assembly checkpoint (SAC) signals (Ohi et al., 2004; Vader et al., 2007). The function of Aurora kinases in rice are unknown so far. As phosphorylation of histone H3 at Ser-10 by Aurora B is conserved across eukaryotes, H3-pS10 is regarded as a marker for normal Aurora activity in many species as well as in rice (Wang et al., 2012b; Gorbsky, 2015).We tested the distribution of H3-pS10 on chromosomes in wild-type and *osprd2* male meiocytes. In wild type, H3-pS10 was localized alongside chromosome at the beginning of diakinesis and gradually enriched at centromeric regions until early metaphase (**Figure 9A**). In *osprd2*, the H3-pS10 showed similar distribution as in wild type (**Figure 9B**), suggesting that Aurora function was not disturbed, thus MT-kinetochore connection may not be affected in *osprd2*.

### 3.7 OsPRD2 is not required for sister chromatid cohesion SGO1 function in rice

**Figure 10:**
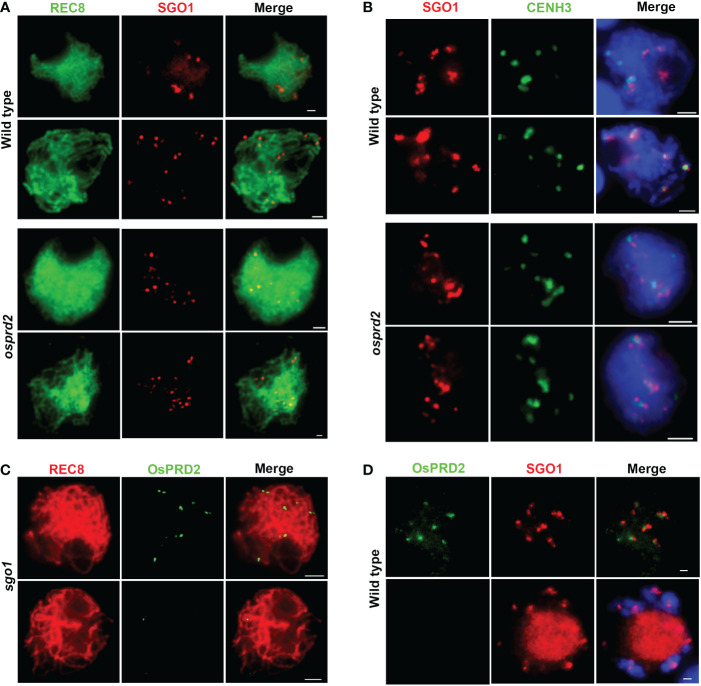
Localization of SGO1 and PRD2 at the centromeric region is independent of each other. **(A, B)** are SGO1 localization in wild type and *osprd2* mutant in zygotene and pachytene; **(C)** is OsPRD2 localization in *sgo1* mutant during zygotene and pachytene; **(D)** is OsPRD2 and SGO1 colocalization in wild type during zygotene and diakinesis. Bar=2μm.

Shugoshins (SGOs) are conserved proteins that are required to protect sister chromatids cohesion adjected to centromere during mitosis and meiosis I. In rice, OsSGO1 is recruited to the centromeric region during leptotene and disassociates from the centromere (Wang et al., 2011). By dual immuno-localization analysis of OsPRD2 and OsSGO1, we found that OsPRD2 colocalized with SGO1 during zygotene (**Figure 10D**). We wondered whether OsPRD2 is involved in recruiting and maintaining the centromeric localization of OsSGO1. We performed immunolocalization using SGO1 and REC8, CENH3 antibody separately to test whether the OsPRD2 mutation influenced OsSGO1 localization (**Figures 10A, B**). We found that in *osprd2*, OsSGO1 localized at the centromere region neighboring to CENH3, similar to its localization in wild type (**Figure 10B**), indicating that OsSGO1 recruitment does not require the presence of OsPRD2.

In addition, we obtained two independent *ossgo1* alleles by CRISPR/Cas9 editing which all caused reading fragment shifting and premature translation termination (**Figure S6A**). The two *ossgo1* mutants showed sterile phenotype and abnormal meiosis progression as previously reported (**Figure S6B**, (Wang et al., 2011). In *ossgo1*, OsPRD2 also normally localized at chromosome during zygotene and released form chromosome during pachytene (**Figure 10C**). These results indicated that OsPRD2 and OsSGO1 localization is independent of each other.

## 4 Discussion

### 4.1 OsPRD2 involves in DSB formation during meiosis

It is well known that in the meiotic DSB machinery, apart from the evolutionarily conserved TOPVIB subunits, other DSB proteins are less conserved and even species specific. Here we demonstrate that the rice ortholog of MEI4/PRD2 has a conserved role in DSB generation.

Mei4 was first found in *S.cerevisiae* as key components involved in meiotic DSB formation, while its counterparts in other organisms were difficult to be recognized due to poor sequence conservation. By analyzing small blocks of conservation, Mei4 homologs were identified in higher eukaryotes (Kumar et al., 2010). In mouse and Arabidopsis, mutants of the MEI4 orthologs are defective in DSB formation (De Muyt et al., 2009; Kumar et al., 2010), suggesting the functional conservation in spite of sequence divergence. In this study, we showed that DSB formation was abolished in *osprd2* evidenced by the absence of γH2AX signals in male meiocytes (**Figure 3A**). Consistent with other DSB defective mutants, later synapsis and recombination were disrupted in *osprd2* (**Figures 3B–F**, **4B–D**). These results indicate the conserved role of OsPRD2 in DSB generation.

In *S. cerevisiae*, the chromosome axis-localized Mer2 recruits Rec114 and Mei4 to assemble the RMM complex (Panizza et al., 2011). Direct interactions between MEI4 and Rec114 orthologs in mouse and Arabidopsis have been reported (Kumar et al., 2018; Vrielynck et al., 2021). However, in Arabidopsis neither AtPRD2 (MEI4 ortholog) nor PHS1(Rec114 ortholog) interact with AtMER2/AtPRD3, but instead both interact with DFO (Vrielynck et al., 2021). In maize and rice, PRD2 also interacts with PRD1 and MTOPVIB (Shi et al., 2021; Wang et al., 2022). But in maize, ZmPRD1 does not interact with either DFO or PHS1, indicating that the DSB protein interaction network is divergent during evolution.

### 4.2 OsPRD2 is not required for bipolar spindle assembly

In Arabidopsis, PRD2/MPS1 has been reported to be involved in spindle assembly during meiosis (Jiang et al., 2009; Walker et al., 2018). Recently, rice OsPRD1 and OsMTOPVIB, as well as maize ZmMTOPVIB have also been found to promote the assembly of the bipolar spindle by maintaining the biorientation of sister kinetochores (Xue et al., 2019; Jing et al., 2020; Shi et al., 2021). Furthermore, OsPRD1 is localized in the centromeric region and interacts with kinetochore and cohesion proteins (Shi et al., 2021). Previous studies showed that OsPRD2 interacts with OsPRD1 and OsMTOPVIB (Shi et al., 2021), implicating that OsPRD2 may also participate in the spindle assembly. However, we found no obvious defects in the bipolar spindle assembly and biorientation of sister kinetochores in *osprd2* male meiocytes (**Figure 6**), suggesting a function divergence with its Arabidopsis ortholog and its interacting proteins in rice in the assembly of bipolar spindle. The functional divergence was also reflected by differences in the chromosome localization of these proteins. OsPRD1 localized at the centromere region from pachytene to diplotene (Shi et al., 2021). By contrast, our results showed that OsPRD2 localized at centromere from zygotene and disappeared from chromosome at early pachytene (**Figure 7A**). In addition, the normal function of OsSDS and CRC1 was required for the centromere localization of OsPRD2 but not OsPRD1 (**Figure 8A**, Shi et al., 2021). A very recent study demonstrated that in maize ZmPRD1 is dispensable for bipolar spindle during meiosis (Wang et al., 2022). These results suggest that the involvement of DSB proteins in spindle assembly is divergent in different species.

### 4.3 OsPRD2 may play a role in centromere function during rice meiosis

The centromeric localization of OsPRD2 foci suggests that it may be required for proper centromeric function. One of the primary functions of the centromere is to assemble the kinetochore, a protein structure that interacts with the spindle and connects the chromosomes to the microtubule ends, a process that is crucial for chromosome segregation into daughter cells during mitosis and meiosis (Allshire and Karpen, 2008). Aurora B acts as one of the Spindle assembly checkpoint (SAC) components, which are recruited to unattached kinetochores to delay chromosome segregation until all chromosomes are correctly attached to the bipolar spindle. During mitosis, in metazoans, Aurora B centromeric localization depends on phosphorylation of Thr-3 on histone H3 (H3T3ph), while active Aurora B then phosphorylates Ser-10 of histone H3 (H3S10ph), which in turn then affects chromosome condensation and segregation (Yamagishi et al., 2010). We observed normal H3S10 signals and no significant defects in spindle attachment in *osprd2* meiocytes (**Figure 9**), suggesting that OsPRD2 may not be required for proper kinetochore assembly and function.

Another function of the centromere is to prevent premature separation of sister chromatids during meiosis I. Cohesion along the chromosome arms were released before anaphase I, while cohesion around the centromeres must be maintained until sister chromatids separated at meiosis II (Watanabe, 2005). No difference in SGO1 localization and abnormalities in sister chromatid separation were found in *osprd2* meiocytes (**Figures 10A, B**), indicating that OsPRD2 may not function in the centromere cohesion protection.

OsPRD2 localized at centromere from zygotene to early pachytene, suggesting that it may be involved in the early meiotic steps. It has been shown that centromere interaction and pairing at early prophase I play an important role in promoting homologous chromosome paring and synapsis (Da Ines and White, 2015, Zhang and Han, 2017). The centromere becomes condensed during homologous chromosome pairing. However we noticed that in *osprd2* meiocytes centromeric condensation was delayed (**Figure S3**). It is possible that a less condensed centromere might interfere with homologous pairing, the progression of synapsis or increase the frequency of aberrant recombination events at centromeric as well as pericentromeric regions. Unfortunately it is not clear if in this case these processes were affected, as they were fully blocked by the absent of DSBs in the mutant. Obtaining weak alleles with remaining bivalents may be useful to further understand the role of OsPRD2 in centromere function.

## Data availability statement

The original contributions presented in the study are included in the article/**Supplementary Material**. Further inquiries can be directed to the corresponding author.

## Author contributions

WL and CW designed this research project. SQ, CW, JZ, and MF performed most of the experiments. XC did rice tissue culture work. CW and WL analysis the data and wrote the manuscript. All authors contributed to the article and approved the submitted version.
