# Supplementary material for: OsPRD2 is essential for double-strand break formation, but not spindle assembly during rice meiosis
Source: Front Plant Sci. 2023 Jan 13;13:1122202. doi: 10.3389/fpls.2022.1122202 (PMC9880466; doi:10.3389/fpls.2022.1122202)
Supplement: Supplementary file 1 [file DataSheet_1.pdf]

Supplementary Figures

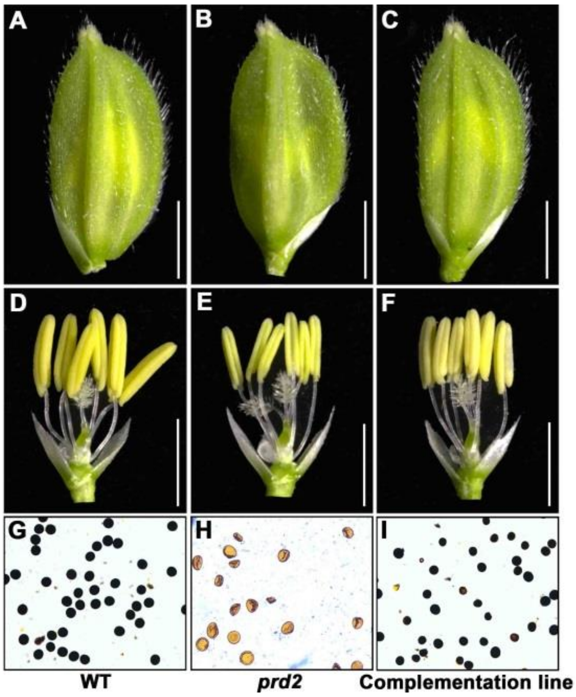

Supplemental figure 1. *OsPRD2* rescue *osprd2* mutant sterile phenotype.

A to C spikelet; D to F inner flower organ; G to I I2-KI staining; A, D and G were wild type; B, E and H were *osprd2* mutant; C, F and I were complementation line. Bar=2mm.

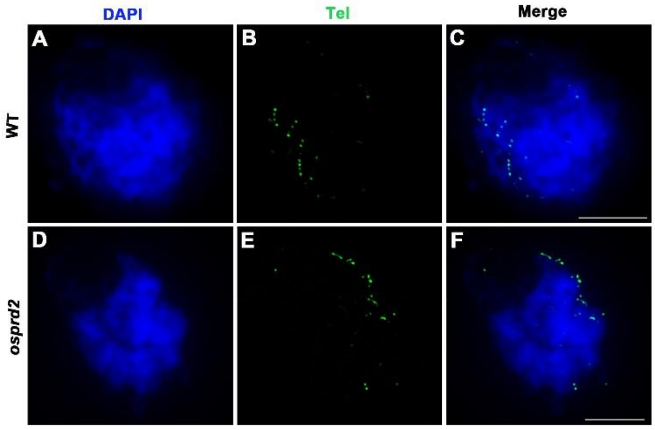

Supplemental figure 2. Telomere bouquet well formed in *osprd2* mutant.

A to C were wild type leptotene; D to F were *osprd2* mutant leptotene; A and D DAPI signal; B and E telomere signal; C and F merge images. Bar=5 μm.

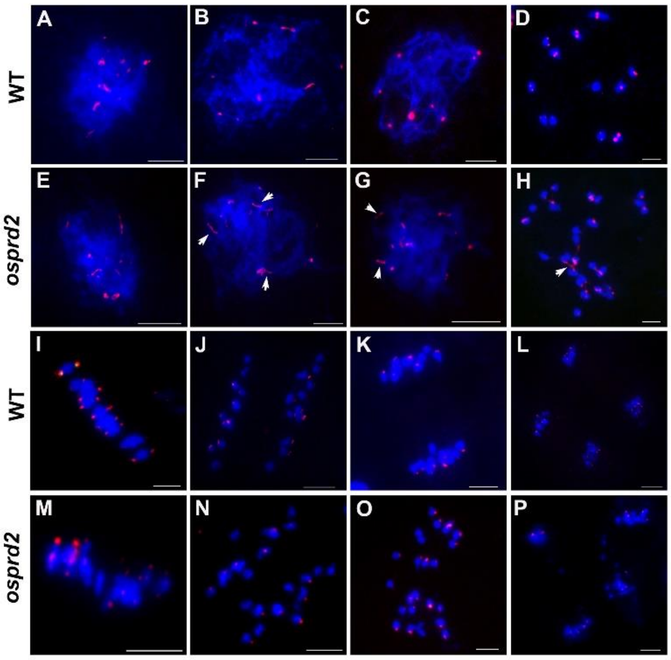

Supplemental figure 3. Elongated centromere signals in *osprd2*.

A to D and I to L were wild type; E to H and M to P were *osprd2* mutant; A and E leptotene; B and F zygotene; C and G pachytene; D and H diakinesis; I and M metaphase I; J, K, N and O anaphase I; L and P telophase II; Bule were DAPI signals; Red were centromere signals. Arrows indicated elongated centromere signals in *osprd2*. Bar=5 μm.

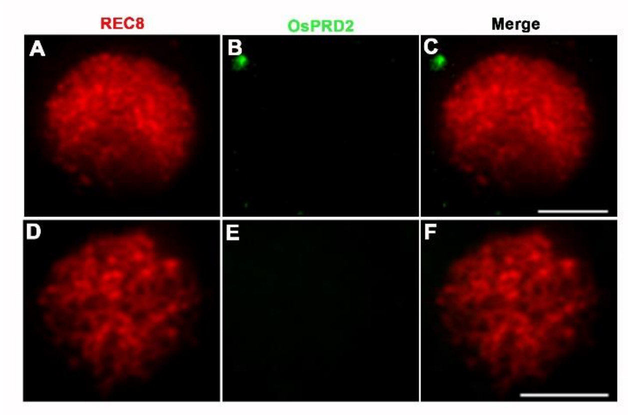

Supplemental figure 4. OsPRD2 localization in *osprd2* mutant

A to C were leptotene; D to F were zygotene; Bar=2 μm.

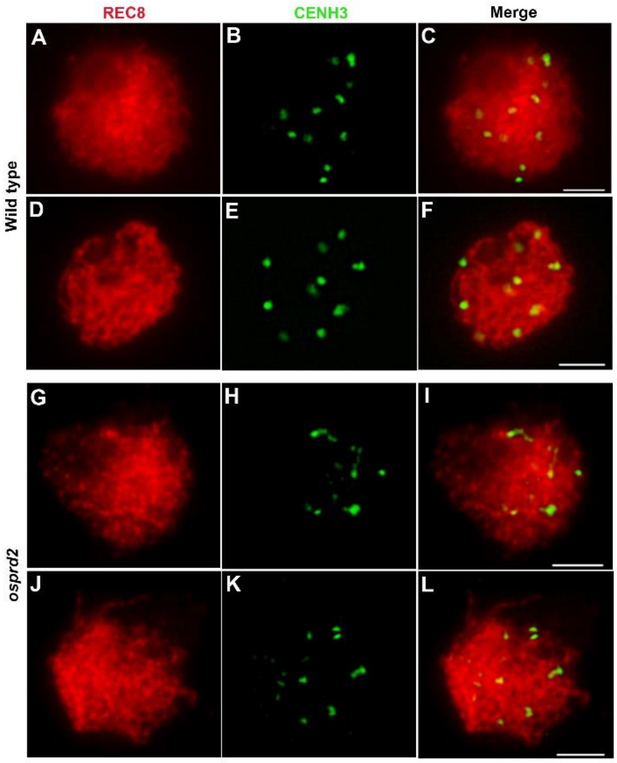

Supplemental figure 5. CENH3 localization in *osprd2* mutant

A to F were wild type; G to L were *osprd2* mutant. A to C and G to I were leptotene; D to F and J to L were zygotene. Bar=2 μm.

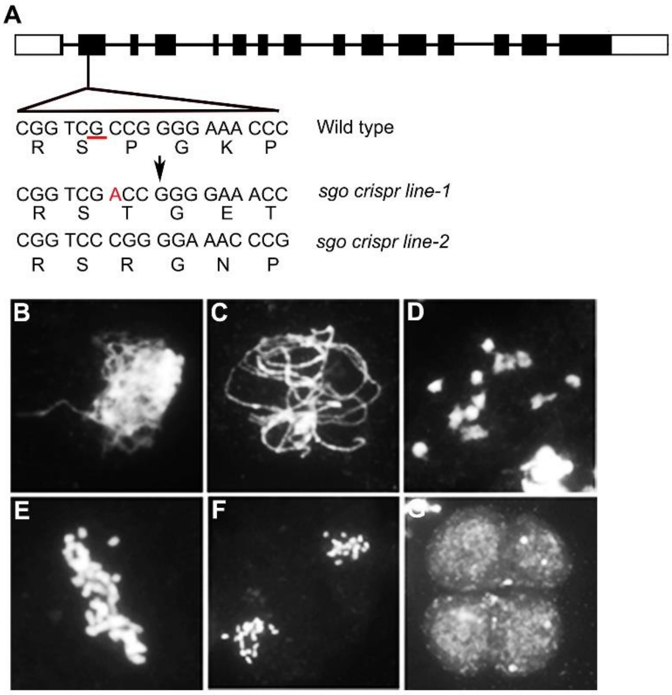

Supplemental figure 6. *SGO1* CRISPR line information and meiosis phenotype.

A was *SGO1* CRISPR line information. Line 1 had an A insertion in the second exon which marked by red color, line 2 had an G deletion in the second exon and marked under red line, these two CRISPR line all leading to reading fragment shaft and translated unmatured protein. B-G is DAPI staining for meiosis process of the *SGO1* CRISPR line, line 1 and line 2 shows similar phenotype.
