# Supplementary material for: OsPRD2 is essential for double-strand break formation, but not spindle assembly during rice meiosis
Source: Front Plant Sci. 2023 Jan 13;13:1122202. doi: 10.3389/fpls.2022.1122202 (PMC9880466; doi:10.3389/fpls.2022.1122202)
Supplement: Supplementary file 2 [file Table_1.docx]

**Supplemental table 1. Primers Used in This Study.**

| **Primer name** | **Sequences** | **Objective** |
| --- | --- | --- |
| PRD2-TXT-F | GAGTAGTTTGATGGGTGGGT | *Osprd2* genotyping |
| PRD2-TXT-R | ATTAAATGTTCAGTCGTGGC | *Osprd2* genotyping |
| PRD2-HB-F | CGGTACCCGGGGATCCAGGCCAGTTTCCGATGATGA | Complementary |
| PRD2-HB-R | ATTCGAGCTGGTCACCGGCAAATGGACAAATGGGGC | Complementary |
| PRD2-Ab-F | AAGGATCCATGGCTCCTCCCGCCTCC | Antibody |
| PRD2-Ab-R | AACCCGGGATTGCTATGCATTTCAGGG | Antibody |
| SGO1-crispr-1F | TAGGTCTCCCTTCGGTCGCCGGTTTTAGAGCTAGAA | *SGO1* CRISPR |
| SGO1-crispr-1R | CGGGTCTCAGAAGGCTGCCACTGCACCAGCCGGG | *SGO1* CRISPR |
| SGO1-crispr-2F | TAGGTCTCCGCTCAAAGTAAAGTTTTAGAGCTAGAA | *SGO1* CRISPR |
| SGO1-crispr-2R | CGGGTCTCAGAGCACGGCTATTGCACCAGCCGGG | *SGO1* CRISPR |
| SGO1-TXT-1F | AAAAATCGTATCCCCTAGAT | Identification for *sgo1* |
| SGO1-TXT-1R | CAAACATTGACACAAGAAT | Identification for *sgo1* |
| SGO1-TXT-2F | TGAAGTCAGCAGAGTAGAG | Identification for *sgo1* |
| SGO1-TXT-2R | GACTGCATACCAATATCTTT | Identification for *sgo1* |
